# Quantifying Airway Invasion and Pharyngeal Residue in Patients with Dementia

**DOI:** 10.3390/geriatrics4010013

**Published:** 2019-01-16

**Authors:** Ashwini M. Namasivayam-MacDonald, Luis F. Riquelme

**Affiliations:** 1 Department of Communication Sciences and Disorders, College of Education and Health Sciences, Adelphi University, Garden City, New York, NY 11530, USA; 2 Department of Speech-Language Pathology, School of Health Sciences and Practice, New York Medical College, Valhalla, New York, NY 10595, USA; luis_riquelme@nymc.edu; 3 Center for Swallowing & Speech-Language Pathology, Department of Neurosciences, NewYork-Presbyterian Brooklyn Methodist Hospital, Brooklyn, New York, NY 11215, USA

**Keywords:** deglutition, dementia, assessment, swallowing, dysphagia, residue, aspiration, penetration

## Abstract

Previous research has begun to elucidate the physiological impairments associated with dysphagia in patients with dementia, but in order to select the most appropriate targets of intervention we need to better understand consequences of dysphagia. The purpose of this study was to quantify penetration, aspiration, and residue in people with dementia, and confirm if residue was associated with airway invasion on subsequent swallows. Videofluoroscopy clips of sips of thin and extremely thick liquid barium from 58 patients with dementia were retrospectively analyzed. Ratings of swallowing safety, using the Penetration–Aspiration Scale (PAS), and efficiency, using Normalized Residue Ratio Scale in the valleculae (NRRSv) and pyriform sinuses (NRRSp), were made on all swallows. Over 70% of both thin and extremely thick liquid swallows were found to be safe (PAS < 3). Results also revealed that residue was generally more common in the valleculae. However, the proportion of thin liquid swallows with significant NRRSp that were unsafe on the subsequent swallow was significantly greater than the proportion of swallows with significant NRRSp that were safe on the subsequent swallow. As such, there was a 2.83 times greater relative risk of penetration–aspiration in the presence of thin liquid pyriform sinus residue. Future research should determine the impaired physiology causing aspiration and residue in this population.

## 1. Introduction

Dementia is one of the main causes of disability later in life, ahead of cancer, cardiovascular disease, and stroke [1]. There are an estimated 5.3 million Americans over the age of 65 currently living with dementia and by 2050, this number could rise as high as 16 million [2]. Total health care system costs and out of pocket costs of caring for people with dementia in the United States were US$259 billion in 2016, and are projected to quadruple by 2050 [2]. Dysphagia (i.e., swallowing difficulties) is a known comorbidity of dementia, with up to 93% of people with dementia presenting with some type of swallowing impairment [3]. There are several consequences of dysphagia, including malnutrition, dehydration, weight loss, and aspiration pneumonia, all of which could lead to death [4,5,6]. While the literature provides several reports of dysphagia in people living with dementia, we still lack a comprehensive understanding of the nature of the impairment.

Anecdotal clinical reports and select literature has focused on the oral phase swallowing of those living with dementia; that is what happens directly within the mouth. One such study reported that participants with dementia presented with rapid and compulsive eating and large bolus sizes [7]. This and similar reports have led to the use of texture-modified foods for managing dysphagia in this population. This food is often unpalatable, may influence amount of oral intake, and may reduce quality of life. Some researchers have also begun to describe dysphagia in people living with dementia using the gold-standard method for detecting swallowing impairments, videofluoroscopy swallowing studies (VFSS; i.e., dynamic swallow X-rays). These studies have reported that the swallowing impairments in dementia are characterized by prolonged swallow durations [8], delayed pharyngeal initiation [9], decreased epiglottic inversion [10,11], reduced hyolaryngeal movement [11,12], and inadequate clearance of the pharynx [13]. While this has helped both the clinical and research communities to begin to understand dysphagia in those with dementia, researchers have yet to quantify these impairments so that comparisons to healthy older adults can be made and disease progression can be tracked. In order to determine the best approaches to treat dysphagia in people living with dementia, we must first establish a better understanding of the underlying physiological impairments.

In general, when we evaluate swallowing impairments, two primary functional concerns are being assessed: (1) the ability to swallow safely, without material entering the airway (“penetration”, when material stays above the vocal folds, and “aspiration”, when material touches or passes the vocal folds); and (2) the ability to swallow efficiently, without leaving residue behind in the pharynx [14]. With an understanding of these consequences of dysphagia, we can then begin to seek out the underlying physiological impairments that are at play in order to develop appropriate rehabilitation plans. The objectives of the current study were to quantify and critically analyze the incidence of penetration, aspiration and post-swallow residue by bolus viscosity in a retrospective sample of patients with dementia, and determine if aspiration was related to post-swallow residue in this population.

## 2. Materials and Methods

Data from this study were extracted from a retrospective clinical archive of videofluoroscopy swallowing studies. Institutional Review Board approval was gained from both NewYork-Presbyterian Brooklyn Methodist Hospital and Adelphi University. The inclusion criteria for the database was that patients had a medical diagnosis of dementia, although the type and severity of the diagnosis is unknown. Recordings in the database come from 58 patients from an acute care hospital. The mean age of the patients at the time of the VFSS was 84.9 (range: 67–100); 26 were female, and 32 were male. All studies were conducted using a Kay Pentax Digital Swallow Workstation recording system, with the fluoroscope in lateral view at 30 pulses per second, and were captured and recorded at 30 frames per second. Standardized 40% weight/volume thin liquid barium from Varibar (Bracco Industries, NJ, USA) (International Dysphagia Diet Standardisation Initiative [IDDSI] level 0) was used for all thin liquid swallowing tasks and EZ-HD powder barium mixed with pudding in a 1:2 ratio (via 5 mL teaspoon) (IDDSI level 4) was used for all extremely thick liquid swallows. The exact volume of the liquid boluses administered was not documented, given that these were clinical videos and natural sip sizes were encouraged, but data extraction for this study was restricted to clips of single sip or teaspoon amounts (continuous cup and straw drinking were excluded).

The clinical VFSS were first spliced into bolus-level clips using Corel Video Studio Pro, and were assigned an alphanumeric code. For each of the thin liquid and extremely thick liquid bolus-level clips available in the dataset, the number of subswallows was recorded. These bolus-level clips contained the swallowing behaviors and swallowing sequence elicited after providing the patient with a single bolus trial. A subswallow was considered to be a single swallow within the swallow sequence captured within the bolus-level clips. In total, 393 thin liquid and 237 extremely thick subswallows were identified. Trained raters went on to further identify each subswallow as non-terminal or terminal swallow in the swallow sequence. The bolus-level clips were then subjected to a variety of measurements, including evaluation of the Penetration–Aspiration Scale (PAS) score [15] and a Normalized Residue Ratio Scale (NRRS) score [16]. The PAS allows one to assess the degree to which the bolus entered the airway and how the patient reacted to the bolus entering the airway, and assigns a score accordingly. The NRRS is an anatomically referenced scale for capturing residue severity using pixel-based measurements, and is primarily used to objectively quantify residue in the vallecuale (NRRSv) and pyriform sinuses (NRRSp). The NRRS measures were taken on a single video frame within a subswallow, where the hyoid was at its most inferior position, the epiglottis had just returned to its vertical position, and once the pharynx had relaxed postswallow [16].

All ratings were completed by a group of trained raters. In order to calculate intra-rater reliability, 20% of ratings were randomly selected and rated in duplicate, unbeknownst to the rater. Another 20% of the ratings were also randomly selected (different from the aforementioned selections) and were rated by a second rater to determine interrater reliability. Once all ratings were completed, reliability measures (two-way mixed intraclass coefficients for consistency [ICCs]) were calculated using SPSS for the NRRSv, NRRSp, and Penetration–Aspiration Scale scores. All reliability measures were found to be “excellent” (ICC > 0.75) [17].

**Figure 1:**
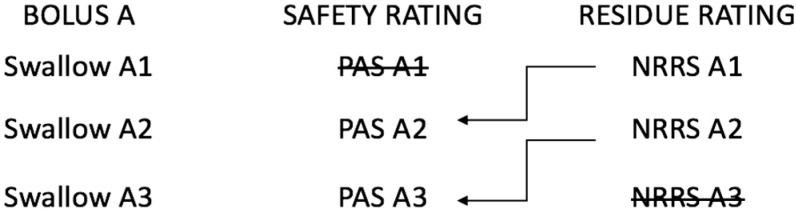
Schematic illustrating a hypothetical three swallow sequence of a single bolus (Bolus A), where the safety ratings from current swallows are analyzed along with residue ratings from previous swallows. PAS: Penetration–Aspiration Scale; NRRS: Normalized Residue Ratio Scale. Schematic is adapted with permission from: Molfenter SM, Steele CM. The Relationship Between Residue and Aspiration on the Subsequent Swallow: An Application of the Normalized Residue Ratio Scale. *Dysphagia* **2013**, *28*, 494–500.

Figure 1 provides a schematic of our secondary objective research question regarding how post-swallow residues predicts airway invasion on the subsequent swallow. Essentially, the PAS score for subswallow two in a given swallow sequence was analyzed with NRRSv and NRRSp scores for subswallow one, and so on and so forth. In order to determine the subswallow clips for which the amount of residue was deemed to be concerning, it was necessary to establish a binary cut-point for residue presence/absence. This was done by comparing our data to the normative values from a healthy sample of adults, where the mean NRRSv score was 0.03 (95% confidence interval (CI): 0.01–0.04) and the mean NRRSp score was 0.00 (95% CI: 0.00–0.01) [18]. The upper confidence interval boundaries for both of these scores were used as cut-points for our dataset. Therefore, any NRRSv score above 0.04 and any NRRSp score above 0.01 were considered to indicate more residue than is considered normal, respectively. We also set an operationally defined binary cut-point for penetration–aspiration of concern. PAS scores of 1 (no penetration or aspiration) and 2 (high transient penetration) were considered to be functional [19,20] and are hereafter referred to as “safe” swallows. A PAS score greater than 2 will be hereafter referred to as “unsafe”.

Descriptive statistics were used to describe the frequency of PAS scores and report the mean NRRSv and NRRSp for non-terminal and terminal swallows. Generalized estimating equations were used to explore the differences in residue by bolus viscosity. We then examined the relationship between pre-swallow residue of concern (i.e., above normal levels of residue remaining from the previous swallow of the same bolus) and impaired swallow safety (PAS scores of 3 or higher) on the subsequent swallow. This relationship was explored for both the valleculae and pyriform sinuses. Comparisons were conducted using chi-squared statistics, with an alpha criterion for significance set at *p* ≤ 0.05. All statistical analyses were conducted using IBM SPSS Statistics for Macintosh, Version 25.0 (IBM Corporation, Armonk, NY, USA).

## 3. Results

### 3.1. Incidence of Penetration, Aspiration, and Residue

Table 1 gives the overall distribution of PAS scores according to bolus viscosity for the entire dataset. More than half of all swallows (55% of thin liquid swallows and 64% of extremely thick liquid swallows) did not invade the airway at all. An additional 15% and 10% of swallows respectively resulted in material entering the airway with immediate ejection before the bolus reached the vocal folds (PAS level of 2). There were very few occurrences of unsafe swallows, with the PAS level of 6 being the least commonly occurring level.

Table 2 provides the overall distribution of NRRSv and NRRSp scores according to swallow position in the sequence and bolus viscosity. Of the 122 nonterminal thin liquid swallows present in the dataset, 92 (75%) surpassed the normal levels of vallecular residue found in healthy adults and 68 (56%) surpassed the normal levels of pyriform sinus residue. A total of 81% (99 of 122) of nonterminal thin liquid swallows resulted in residue in the valleculae and/or the pyriform sinuses. There were a total of 271 terminal thin liquid swallows, of which 161 (59%) had more than normal amounts of vallecular residue and 87 (32%) had more than normal amounts of pyriform sinus residue. One hundred and seventy-nine (66%) terminal thin liquid swallows resulted in more than normal amounts of residue in the valleculae and/or the pyriform sinuses. Of the 393 total thin liquid swallows, 253 (64%) had significant vallecular residue, 155 (39%) had significant pyriform sinus residue, and 278 (71%) had either vallecular and/or pyriform sinus residue. There were 113 thin liquid swallows (of 122 nonterminal swallows; 93%) that prompted an immediate clearing swallow. Twenty-one (19%) clearing swallows were effective at clearing above normal levels of vallecular residue, and 24 (21%) clearing swallows were effective at clearing above normal levels of pyriform sinus residue.

When evaluating the nonterminal extremely thick liquid swallows, it was found that 54 of the 67 (81%) swallows had more than normal amounts of residue in the valleculae and 29 of the 67 (43%) swallows had more than normal amounts of residue in the pyriform sinuses. Approximately 84% (56 out of 67) nonterminal extremely thick liquid swallows resulted in some type of residue. There were a total of 170 terminal extremely thick liquid swallows, of which 103 (61%) had more than normal amounts of vallecular residue and 43 (25%) had more than normal amounts of pyriform sinus residue. One hundred and nine (64%) extremely thick liquid swallows resulted in more than normal amounts of residue in either the valleculae and/or pyriform sinuses. Of the 237 extremely thick liquid swallows, 157 (66%) had significant vallecular residue, 72 (30%) had significant pyriform sinus residue, and 165 (70%) had either vallecular and/or pyriform sinus residue. There were 30 extremely thick liquid swallows (of the 67 nonterminal swallows; 45%) that prompted an immediate clearing swallow. Seven (23%) clearing swallows were effective at clearing above normal levels of vallecular residue, and seven (23%) clearing swallows were effective at clearing above normal levels of pyriform sinus residue.

**Figure 2:**
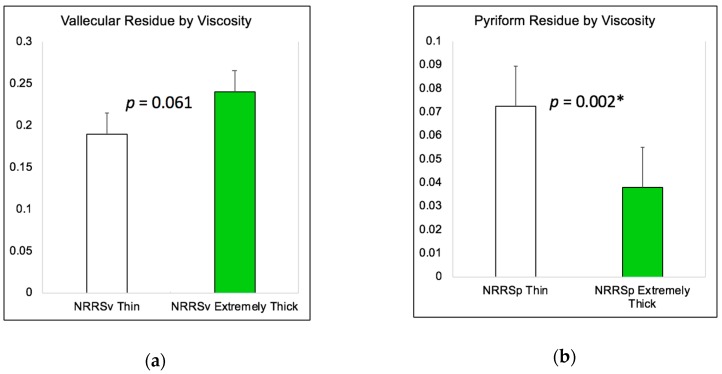
(**a**) Bar graph depicting in differences in vallecular residue (NRRSv) by bolus viscosity; (**b**) Bar graph depicting in differences in pyriform sinus residue (NRRSp) by bolus viscosity. * *p* ≤ 0.05.

Figure 2a illustrates the differences in vallecular residue (NRRSv) according to bolus viscosity, and Figure 2b illustrates the differences in pyriform sinus residue (NRRSp) according to bolus viscosity. As can be seen in these figures, there was no significant change in vallecular residue based on stimuli (χ^2^ (1) = 3.50, *p* = 0.061) yet significantly worse pyriform sinus residue with thin liquids compared to extremely thick liquids (χ^2^ (1) = 9.51, *p* = 0.002).

### 3.2. The Relationship between Residue and Aspiration on the Subsequent Swallow

The odds of unsafe secondary swallows in the presence of significant residue were calculated based on the thresholds for residue based on a sample of healthy adults [18]. There were 59 (65%) thin liquid swallows that resulted in above normal levels of vallecular residue where the subsequent swallow was considered to be safe, and 32 (35%) swallows where the subsequent swallow was unsafe. Similarly, there were 41 (60%) thin liquid swallows resulting in above normal levels of pyriform sinus residue where the subsequent swallow was safe, and 27 (40%) swallows where the subsequent swallow was unsafe. We found no relationship between pre-swallow residue in either one or both pharyngeal spaces and swallowing safety on the immediately occurring subsequent clearing swallow for thin liquids (χ^2^ (1) = 2.33, *p* = 0.127) nor extremely thick liquids (χ^2^ (1) = 1.58, *p* = 0.208). However, when analyzed separately by bolus location, only thin liquid pre-swallow residue in the pyriform sinuses demonstrated a significant relationship with subsequent swallow safety. A significantly greater proportion of swallows displaying thin liquid pre-swallow pyriform sinus residue of concern were found to be unsafe (73%) compared to the proportion seen in swallows without thin liquid pre-swallow pyriform sinus residue of concern (27%) [χ^2^ (1) = 6.09, *p* = 0.013; OR: 2.83, 95% CI: 1.22–6.57]. There were no significant relationships found between unsafe swallows and thin liquid vallecular residue (χ^2^ (1) = 3.64, *p* = 0.057), unsafe swallows and extremely thick liquid vallecular residue (χ^2^ (1) = 0.64, *p* = 0.424), nor unsafe swallows and extremely thick liquid pyriform sinus residue (χ^2^ (1) = 0.0019, *p* = 0.966). There was a total of 40 (74%) extremely thick liquid swallows that resulted in above normal levels of vallecular residue where the subsequent swallow was considered to be safe, and 14 (26%) swallows where the subsequent swallow was unsafe. Similarly, there were 22 (76%) extremely thick liquid swallows resulting in above normal levels of pyriform sinus residue where the subsequent swallow was safe, and 7 (24%) swallows where the subsequent swallow was unsafe.

## 4. Discussion

The current study set out to quantify penetration, aspiration and residue in a sample of patients with dementia, and determine if residue was associated with airway invasion on subsequent swallows. Through this retrospective analysis, we found that the majority of swallows (70% of thin liquid swallows and 74% of extremely thick liquid swallows) were deemed to be safe. Furthermore, residue was a more common sign of swallowing impairments in this population, with around 70% of thin and extremely thick liquid swallows resulting in significant residue in either the valleculae and/or pyriform sinuses. We also found that the relative risk of an unsafe swallow was three times greater in the presence of thin liquid pyriform sinus residue.

The finding of relatively few unsafe swallows is supported by previous work that found no aspiration on thin liquids in a sample of 24 patients with Alzheimer’s disease [12]. There is also very little penetration and aspiration expected in healthy older adults [21]. This suggests that mechanisms contributing to swallowing safety, including adduction of the true vocal folds, approximation of the false vocal folds, epiglottic inversion, and anterior movement of the arytenoids to approximate the base of the epiglottis [22,23,24,25], remain relatively intact with a dementia diagnosis. When the results of the present study are compared to results of a similar study evaluating residue in a sample of patients with neurogenic dysphagia (i.e., patients with a medical diagnosis of primarily stroke or brain injury), we find that patients with dementia present with more vallecular residue on thin liquid boluses [26]. This study by Molfenter and Steele [26] found that patients with neurogenic dysphagia had a mean NRRSv score of 0.09 ± 0.17 and a mean NRRSp score of 0.19 ± 0.4 on nonterminal swallows, compared to the present findings in patients with dementia who had scores 0.32 ± 0.32 and 0.15 ± 0.34, respectively. For terminal, thin liquid swallows, adults with neurogenic dysphagia had NRRS scores of 0.03 ± 0.11 in the valleculae and 0.04 ± 0.14 in the pyriform sinuses, whereas our sample of adults with dementia had scores of 0.16 ± 0.25 and 0.04 ± 0.09, respectively. Interestingly, patients with neurogenic dysphagia had a 2.07 times greater risk of an unsafe swallow in the presence of significant thin liquid residue in the valleculae, whereas patients with dementia had a greater risk of aspiration when residue was found in the pyriform sinuses. However, one should note that the relationship found between unsafe swallows and thin liquid vallecular residue neared significance, so with a larger sample size risk of aspiration might have also been greater in the presence of thin liquid vallecular residue. The differences in findings between the current dementia sample and the neurogenic dysphagia sample likely speak to the differences in swallowing pathophysiology. Given the heterogeneity of the sample of patients with neurogenic dysphagia due to probable differences in type, size and location of injury, it is difficult to surmise the mechanisms resulting in impaired physiology. Similarly, a systematic review detailing oropharyngeal dysphagia in different types of dementia suggests that the nature of oropharyngeal dysphagia varies according to the type of dementia [27], making it also difficult to understand why patients with simply a diagnosis of “dementia” have more vallecular residue. For example, in the review, the authors describe how individuals with Alzheimer’s Disease present with primarily oral phase deficits, such as loss of food from the oral cavity and difficulty chewing. There was also mention of a delayed swallow trigger in this subset of patients, as well as reduced hyolaryngeal elevation and aspiration as the disease progresses. In contrast, the review suggested that patients with frontotemporal dementia most often present with behavioral feeding problems, delayed pharyngeal swallow response, and incomplete bolus clearance resulting in post-swallow residue. Because the mechanisms behind pharyngeal residue are still being researched, it is possible that many of the pharyngeal phase deficits mentioned for either of the dementia sub-types could lead to pharyngeal residue.

One study that has been conducted on pharyngeal residue in the elderly proposes that it is most often the result of poor tongue driving forces, reduced pharyngeal shortening, and reduced pharyngeal constriction [28]. These authors concluded that vallecular residue is most closely associated with tongue driving forces and pyriform sinus residue is most closely associated with limited pharyngeal shortening [28]. Another study by Stokely and colleagues [29] suggested that those with pharyngeal residue tend to have a larger pharyngeal area at rest, which could contribute to reduced pharyngeal constriction during the swallow. Further research found that increasing viscosity leads to more pharyngeal shortening, less constriction, and more residue in healthy older adults [30]. In our sample of patients with dementia, more residue (i.e., higher NRRS scores) was evident for extremely thick liquids in the valleculae, but this was not the case for the pyriform sinuses, which was surprising given that healthy older adults presented with more residue in both spaces. Given the large percentage (93%) of immediate clearing swallows following thin liquid boluses, one might be led to believe that sensory impairments are unlikely contributors to increased residue in the pharynx. However, the percentage of clearing swallows was much lower for extremely thick liquid boluses (45%) and the effectiveness of clearing swallows for both types of boluses was limited. As such, the presence of sensory impairments warrants further investigation in this population. In order to truly elucidate the reasons for residue in patients with dementia, future research should consider analyzing the physiological mechanisms, including timing, kinematics and sensation, suggested to contribute to residue in this population in order to create targeted intervention protocols.

While the present study quantifies the most common consequences of dysphagia—penetration, aspiration, and residue—there are some limitations that should be acknowledged. Firstly, since this was a retrospective analysis of patient videos, radiation exposure considerations were made that lead to limited monitoring of events post-swallow. Therefore, it is possible that delayed instances of airway invasion took place while the video was off between swallows, and this was not captured in the current analysis. It is also important to note that non-cued and cued swallows were not controlled for in this dataset. Past research with a healthy aged population has indicated that verbal cues to initiate a swallow result in altered swallow patterns [31]. However, is understood that for this clinical population it is sometimes clinically necessary to verbally cue a swallow (i.e., to elicit a swallow on command). We also do not know the average sip size taken for each type of bolus. The current study was also unable to differentiate between types of dementia due to the nature of information provided in each patient’s medical records. Previous research has confirmed that dysphagia differs based on type of dementia [27], so future work should attempt to parse out these differences in more detail and account for the presence of other chronic medical conditions that may influence swallowing. Lastly, the current study did not report on any timing or kinematic measures to determine the mechanism contributing to unsafe swallow and residue. It also does not address differences in swallow physiology by severity of dementia, which should be considered in future studies given that changes are also expected as severity increases in the presence of any neurodegenerative disease. As this population continues to grow across the globe, efforts must be undertaken to better understand the pathophysiology of changes in the swallow mechanism in order to develop interventions that will maintain good nutrition and quality of life, as well as reduce health risks such as malnutrition, dehydration, and aspiration.

## 5. Conclusions

The current study provides additional data on the pathophysiology of the swallow in persons with dementia. Of interest, more than half of the swallows analyzed did not result in airway invasion, and the majority of those that did were considered safe, as the material was ejected before reaching the vocal folds. Also noted was that over 60% of non-terminal and terminal swallows presented significant residue in either the vallecuale or pyriforms when compared to healthy normals. Lastly, no relationship was identified between pre-swallow residue and subsequent clearing swallow safety for either consistency. However, when bolus residue location was analyzed separately, only thin liquid pre-swallow residue in the pyriform sinuses demonstrated a significant relationship with subsequent swallow safety (73% were considered unsafe). The relevance of these findings can be measured in the context of future exploration and understanding of the swallow pathophysiology in persons with dementia. Clinically, these data may serve to influence practice patterns for the recommendation of texture-modified liquids and foods (extremely thick liquids are also considered puree foods in the IDDSI framework, level 4). As previously suggested, this may impact the safety and efficiency of the swallow in this population.

## Figures and Tables

**Table 1:** Distribution of Penetration–Aspiration Scale (PAS) scores according to bolus viscosity.

PAS Score	PAS Description	*n* (%) of Thin Liquid Swallows	*n* (%) of Extremely Thick Liquid Swallows
1	Material does not enter the airway.	216 (55%)	152 (64%)
2	Material enters the airway, remains above the vocal folds, and is ejected from the airway.	59 (15%)	23 (10%)
3	Material enters the airway, remains above the vocal folds, and is not ejected from the airway.	43 (11%)	35 (15%)
4	Material enters the airway, contacts the vocal folds, and is ejected from the airway.	8 (2%)	1 (0.33%)
5	Material enters the airway, contacts the vocal folds, and is not ejected from the airway.	16 (4%)	1 (0.33%)
6	Material enters the airway, passes below the vocal folds, and is ejected into the larynx or out of the airway.	4 (1%)	1 (0.33%)
7	Material enters the airway, passes below the vocal folds, and is not ejected from the trachea despite effort.	20 (5%)	9 (4%)
8	Material enters the airway, passes below the vocal folds, and no effort is made to eject.	27 (7%)	15 (6%)

**Table 2:** Distribution of Normalized Residue Ratio Scale (NRRS) scores for the valleculae (NRRSv) and pyriform sinus (NRRSp) by nonterminal versus terminal swallow within the swallow sequence.

Stimuli	Swallow Status	*n* (%)	NRRSv			NRRSp		
			Mean	SD	95% CI	Mean	SD	95% CI
Thin liquid	Nonterminal	122 (31%)	0.32	0.32	0.26–0.37	0.15	0.34	0.10–0.19
	Terminal	271 (69%)	0.16	0.25	0.13–0.19	0.04	0.09	0.03–0.05
Extremely thick liquid	Nonterminal	67 (28%)	0.41	0.57	0.28–0.55	0.08	0.19	0.04–0.13
	Terminal	170 (72%)	0.21	0.30	0.16–0.25	0.02	0.07	0.00–0.03

SD standard deviation; CI confidence interval.
